# Linking subjective well-being to short-video addiction among college students: the serial mediating roles of alienation and self-control

**DOI:** 10.3389/fpsyt.2026.1892672

**Published:** 2026-08-20

**Authors:** Yang Peng, Ying Cao, Yao Qin, Ting Xiao, Aolei Yang, Yanyun Yuan, Yueyue Meng

**Affiliations:** 1 School of Educational Science, Hunan Normal University, Changsha, China; 2 Counseling Center, Hunan Non-ferrous Metals Vocational And Technical College, Zhuzhou, China; 3 Research Centre for Mental Health Education of Hunan Province, Changsha, China; 4 Cognition and Human Behavior Key Laboratory of Hunan Province, Changsha, China

**Keywords:** subjective well-being, short-video addiction, alienation, self-control, problematic media use, college students

## Abstract

Short-video addiction has become an increasingly important concern among college students, but the psychological processes through which general well-being is associated with problematic short-video use remain insufficiently understood. Drawing on the social compensation perspective and self-control theory, this study examined whether alienation and self-control are associated with the association between subjective well-being and short-video addiction. A total of 1,731 college students from eight universities in Hunan Province completed measures of subjective well-being, alienation, self-control, and short-video addiction. Correlation analyses and structural equation modeling indicated that subjective well-being was negatively associated with short-video addiction. Alienation and self-control each mediated this association, and a significant serial mediation pathway was also found. Specifically, lower subjective well-being was associated with greater alienation, which was further related to weaker self-control and higher levels of short-video addiction. These findings suggest that problematic short-video use among college students may reflect not only excessive media engagement, but also a broader psychological process involving social-emotional disconnection and impaired self-regulation. By integrating the social compensation perspective with self-control theory, this study provides a more comprehensive account of the mechanisms linking subjective well-being to short-video addiction and suggests the potential value of interventions that enhance well-being, reduce alienation, and strengthen self-control among college students.

## Introduction

Short-video platforms have become deeply embedded in young adults’ everyday digital lives, offering rapid access to entertainment, information, and social interaction. National surveys indicate that Chinese college students spend an average of approximately three hours per day on short-video platforms, with Douyin and Kuaishou being the most frequently used (1). Students primarily use short videos for entertainment, information seeking, and social interaction (2, 3). For college students, however, the same design features that make these platforms appealing, including algorithmic recommendation, continuous content streams, and immediate emotional rewards, may also increase the likelihood of excessive and poorly controlled use (4). Short-video addiction generally refers to a maladaptive pattern of short-video engagement characterized by persistent use, difficulty regulating viewing time or frequency, and negative consequences for psychological, academic, and daily functioning (5). Although short-video addiction shares some features with generalized Internet addiction, such as excessive use and loss of control, short-video platforms are distinguished by algorithm-driven content delivery, endless scrolling, and rapid emotional rewards—features that may involve psychological mechanisms not fully captured by research on generalized Internet addiction. Indeed, prior research has demonstrated the theoretical and empirical value of distinguishing between generalized and specific forms of Internet addiction (6). Thus, examining short-video addiction as a separate phenomenon is warranted.

Accumulating evidence has linked problematic short-video use to poorer sleep quality, heightened depressive and anxious symptoms, impaired academic functioning, and reduced self-regulation (7, 8). Recent work has also begun to examine its associations with subjective well-being, social support, personality traits, and self-control, suggesting that short-video addiction may reflect broader difficulties in psychological and social adjustment rather than excessive screen time alone (9, 10). Nevertheless, the psychological mechanisms through which students’ general well-being is associated with problematic short-video use remain insufficiently understood. In particular, less is known about how social-emotional disconnection and self-regulatory capacity may be jointly associated with this association. The present study examined whether alienation and self-control serve as mediating pathways in the association between subjective well-being to short-video addiction among college students.

### Subjective well-being and short-video addiction

Subjective well-being refers to individuals’ overall evaluation of their lives and emotional experiences, encompassing both cognitive judgments, such as life satisfaction, and affective experiences, such as positive and negative affect (11). As a core indicator of psychological adjustment, subjective well-being be related to why some college students are more vulnerable to problematic short-video use. From the perspective of the social compensation model, individuals with lower subjective well-being are more likely to experience dissatisfaction in daily life, weaker interpersonal connection, and insufficient social support (12). Under such conditions, short-video platforms may provide an easily accessible source of distraction, emotional relief, and compensatory gratification (13). Their continuous streams of entertaining and emotionally rewarding content may temporarily alleviate negative affect and satisfy unmet psychological needs; however, repeated reliance on this form of compensation may increase the likelihood of excessive and poorly controlled use (14). This interpretation is consistent with evidence showing that negative emotional states are closely associated with short-video addiction (8). By contrast, students with higher subjective well-being may possess more stable emotional resources, stronger real-life engagement, and less need to seek immediate psychological gratification through short-video platforms (4). These theoretical and empirical considerations suggest that subjective well-being may be negatively associated with short-video addiction among college students.

### Alienation as a social-emotional pathway

Alienation refers to a subjective sense of powerlessness, loneliness, emptiness, and disconnection that arises in individuals’ interactions with their social environment and is often reflected in psychological distance from the self, others, and the surrounding context (15). In the context of college students, alienation may manifest in students’ daily lives—for example, feeling detached from classmates, lacking a sense of belonging to campus life, doubting the meaning of academic goals, or perceiving a gap between oneself and others. These experiences of disconnection are particularly relevant to college students, as they navigate a developmental period characterized by identity exploration, social integration, and academic adjustment (16, 17). This construct offers a useful perspective for understanding how subjective well-being may be linked to problematic short-video use. According to the social compensation hypothesis, individuals who experience difficulties in offline relationships may turn to online environments for emotional relief, social connection, and compensation for unmet interpersonal needs (12). For college students with lower subjective well-being, short-video platforms may therefore provide an accessible way to escape loneliness, dissatisfaction, or perceived social disconnection. However, repeated reliance on short-video content as a compensatory resource may further reduce real-life engagement and may reinforce feelings of alienation.

Prior research has shown that alienation is associated with internalizing problems and maladaptive online behaviors, including Internet addiction and pathological Internet use (18, 19). As a specific form of problematic Internet-related behavior, short-video addiction may similarly be embedded in this broader pattern of social-emotional maladjustment. At the same time, subjective well-being is closely related to social integration, emotional security, and perceived belongingness (20). Students with higher subjective well-being may have more supportive interpersonal relationships and greater psychological resources for coping with social and environmental stress, which may protect them from feelings of alienation (21). Conversely, lower subjective well-being may be accompanied by weaker real-life connection and greater social-emotional disconnection, thereby increasing vulnerability to problematic short-video use (9). Thus, alienation may serve as an important social-emotional pathway through which subjective well-being is associated with short-video addiction among college students.

### Self-control as a self-regulatory pathway

Self-control refers to the capacity to monitor and regulate one’s thoughts, emotions, and behaviors in the service of long-term goals, particularly when immediate temptations or habitual impulses are present (22). This capacity is especially relevant to short-video addiction because short-video platforms are designed to deliver rapid stimulation, immediate rewards, and continuous content exposure. From the perspective of self-control theory, individuals with lower self-control are more likely to prioritize short-term gratification over long-term goals and are therefore more vulnerable to impulsive and addictive behaviors (23). Consistent with this view, empirical studies have shown that lower self-control is associated with more frequent mobile phone checking, greater mobile phone overuse, and higher risk of Internet addiction and excessive short-video use (24).

Self-control may also be related to the association between subjective well-being and short-video addiction. Previous research has identified self-control as an important correlate of subjective well-being (25), and individuals with higher subjective well-being tend to show stronger self-regulatory capacity (26). Positive emotional states may provide psychological resources that support goal-directed behavior, whereas negative affect may impair self-regulation and increase susceptibility to immediate rewards. In line with this reasoning, negative emotions have been shown to weaken self-control and contribute to addictive behaviors by reducing individuals’ ability to regulate impulses (27, 28). By contrast, individuals with higher subjective well-being may derive greater satisfaction from real-life activities and may rely less on short-video platforms for immediate emotional gratification (29). They may also be more likely to use adaptive emotion-regulation strategies that reduce unnecessary conflicts, support long-term goal pursuit, and help maintain self-control resources (30, 31). Taken together, these theoretical and empirical findings suggest that self-control may serve as a self-regulatory pathway through which subjective well-being is associated with short-video addiction among college students.

### The present study

**Figure 1:**
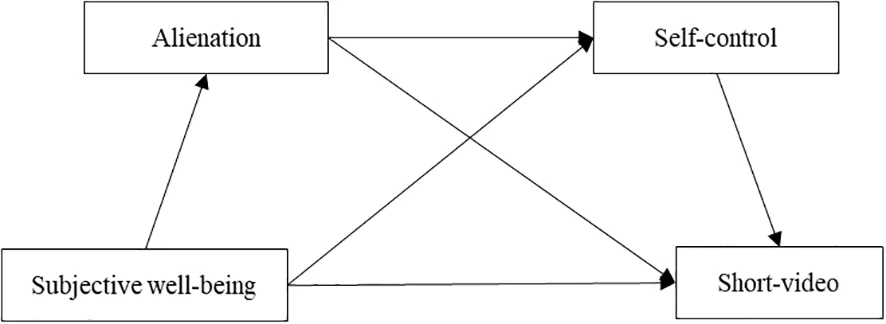
Proposed serial mediation model linking subjective well-being to short-video addiction.

Although previous research has examined subjective well-being, alienation, self-control, and problematic Internet use, these variables have often been studied in separate or partial models. Less is known about how social-emotional disconnection and self-regulatory capacity may jointly associated with college students’ vulnerability to short-video addiction. To address this gap, the present study drew on the social compensation model and self-control theory to propose and test a novel serial mediation model that integrates a social-emotional pathway (via alienation) and a self-regulatory pathway (via self-control). Specifically, we hypothesized a sequential mechanism whereby lower subjective well-being heightens feelings of alienation, which in turn impairs self-control capacity, ultimately increasing the risk of short-video addiction. This integrated framework represents a theoretical advancement over previous studies that have largely examined these pathways in isolation. By focusing specifically on short-video addiction—a distinct and increasingly prevalent form of problematic media use characterized by algorithm-driven, highly immersive, and easily accessible content—this study extends the existing literature beyond generalized Internet addiction. By testing these joint pathways simultaneously in a large sample of college students, this study may offer a more comprehensive understanding of the psychological processes underlying the association between subjective well-being and problematic short-video use. The proposed serial mediation model is presented in **Figure 1**.

## Participants and procedure

A total of 1,800 college students were recruited from eight universities in Hunan Province using a combination of cluster sampling and random sampling. Participants were invited to complete a questionnaire survey assessing subjective well-being, alienation, self-control, and short-video addiction. Questionnaires with substantial missing data, patterned responses, or other indicators of invalid responding were excluded from the analysis. After removing 69 invalid questionnaires, the final analytic sample consisted of 1,731 students, yielding a valid response rate of 96.17%. Among the participants, 818 were male and 913 were female. In terms of academic year, 730 were first-year students, 848 were second-year students, and 153 were third-year students.

The study protocol was reviewed and approved by the Academic Ethics Committee of Hunan Normal University. Before completing the survey, participants received standardized instructions from trained research assistants and were informed of the purpose of the study, the voluntary nature of participation, and the confidentiality of their responses. All participants provided informed consent. The questionnaires were administered in a quiet classroom setting and completed anonymously. The survey took approximately 20 minutes to complete. All returned questionnaires were checked and screened before being entered into the final data analysis.

## Measures

### Subjective well-being

Subjective well-being was assessed using the Chinese version of the Index of Well-Being developed by Campbell (32). This instrument is designed to measure individuals’ general subjective evaluation of their life and emotional experience. It consists of two components: the Index of General Affect, which includes eight items assessing general affective experience, and the Index of Life Satisfaction, which includes one item assessing overall satisfaction with life. All items are rated on a 7-point scale. Following the original scoring procedure, the Index of General Affect is weighted by 1.0 and the Index of Life Satisfaction is weighted by 1.1 when calculating the total score. Higher scores indicate higher levels of subjective well-being. The scale has been widely used in Chinese samples and has demonstrated acceptable psychometric properties. In the present study, Cronbach’s alpha for the scale was 0.92.

### Short-video addiction

Short-video addiction was measured using the Short Video Addiction Scale for College Students developed by Qin Haoxuan et al. (33). This scale was specifically designed to assess problematic short-video use among college students. It contains 14 items covering four dimensions: withdrawal, escape, loss of control, and inefficiency. The withdrawal dimension reflects discomfort or craving when short-video use is restricted; escape captures the tendency to use short videos to avoid negative emotions or real-life stress; loss of control refers to difficulty regulating the frequency or duration of use; and inefficiency reflects negative effects of short-video use on study, work, or daily functioning. Participants rated each item on a 5-point Likert scale ranging from 1 = strongly disagree to 5 = strongly agree. Higher total scores indicate higher levels of short-video addiction. Previous research has supported the reliability and validity of this scale among college students. In the present study, Cronbach’s alpha was 0.93.

### Alienation

Alienation was assessed using the College Students’ Alienation Scale developed by Deng (34). The scale contains 15 items and assesses alienation across four dimensions: self-alienation, environmental alienation, interpersonal alienation, and cultural value alienation. Self-alienation reflects a sense of estrangement from one’s own identity or inner experience; environmental alienation refers to psychological distance from one’s surroundings; interpersonal alienation captures feelings of disconnection from others; and cultural value alienation reflects a sense of detachment from shared values or social norms. Items are rated on a 7-point Likert scale ranging from 1 = strongly disagree to 7 = strongly agree. Higher total scores indicate higher levels of alienation. The scale has shown good reliability and validity in college student samples. In the present study, Cronbach’s alpha was 0.87.

### Self-control

Self-control was measured using the Self-Control Scale originally developed by Tangney et al. (35) and revised for Chinese samples by Tan and Guo (36). The scale consists of 19 items assessing five dimensions of self-control: impulse control, healthy habits, resistance to temptation, focus on work, and moderation in leisure activities. These dimensions reflect individuals’ ability to inhibit impulses, maintain adaptive routines, resist immediate temptations, stay focused on goal-directed tasks, and regulate leisure behaviors. Participants rated each item on a 5-point Likert scale ranging from 1 = strongly disagree to 5 = strongly agree. Higher total scores indicate stronger self-control. To better align the measure with the context of problematic Internet and short-video use, Item 19 was adapted from “I sometimes drink excessively or go online” to “I sometimes go online excessively.” As excessive alcohol consumption was not relevant to the present research focus, this adaptation was intended to enhance the content relevance and face validity of the scale for our specific study population. The revised scale has demonstrated good reliability and validity in previous research. In the present study, Cronbach’s alpha was 0.83.

## Data analysis

All statistical analyses were conducted using SPSS 22.0 and AMOS 23.0. Prior to the main analyses, the returned questionnaires were screened for invalid responses, and the dataset was checked for completeness, accuracy, and response quality. Questionnaires with substantial missing data, patterned responding, or other indicators of invalid responses were excluded from the final analysis. After data screening, descriptive statistics were calculated for all study variables, including means and standard deviations.

Because all variables were assessed using self-report questionnaires, potential common method bias was examined before hypothesis testing. Harman’s single-factor test was conducted to determine whether a single factor accounted for the majority of variance among the measured items. Pearson correlation analyses were then performed to examine the bivariate associations among subjective well-being, alienation, self-control, and short-video addiction.

To evaluate the factorial validity of the measurement model, we conducted a confirmatory factor analysis (CFA) using AMOS 23.0. Latent variables were constructed using item parcels based on the theoretically defined dimensions of each scale. Specifically, subjective well-being was parceled into eight affect items and one life satisfaction item; alienation into four subscale dimensions (self-alienation, environmental alienation, interpersonal alienation, and cultural value alienation); self-control into five dimensions (impulse control, healthy habits, resistance to temptation, focus on work, and moderation in leisure activities); and short-video addiction into four dimensions (withdrawal, escape, loss of control, and inefficiency).

To test the proposed chain mediation model, structural equation modeling was conducted in AMOS 23.0. In the model, subjective well-being was specified as the independent variable, short-video addiction as the dependent variable, and alienation and self-control as mediating variables. Model fit was evaluated using multiple indices, including the chi-square to degrees of freedom ratio, the normed fit index, the comparative fit index, the incremental fit index, and the root mean square error of approximation. The significance of the indirect effects was assessed using a bootstrapping procedure with 5,000 resamples. Mediation effects were considered statistically significant when the 95% confidence interval did not include zero.

## Results

### Preliminary analyses

Before testing the hypothesized mediation model, preliminary analyses were conducted to evaluate the quality and suitability of the data. Given that all study variables were measured using self-report questionnaires, Harman’s single-factor test was performed to assess the potential influence of common method bias (37). The exploratory factor analysis extracted nine factors with eigenvalues greater than 1, and the first unrotated factor accounted for 31.82% of the total variance, which was below the commonly used 40% threshold (38). These results suggest that common method bias was unlikely to seriously affect the interpretation of the findings, supporting the suitability of the data for subsequent correlation and mediation analyses.

### Measurement model

To evaluate the factorial validity of the measurement model, we conducted a confirmatory factor analysis (CFA) using AMOS 23.0. The CFA results indicated an acceptable fit to the data: χ²/df = 14.06, CFI = 0.92, RMSEA = 0.09 (90% CI [0.08, 0.09]). All factor loadings were statistically significant (*p* < 0.001) and ranged from 0.42 to 0.86, with most exceeding the recommended threshold of 0.50 (39). These results indicated that the measurement model adequately represented the latent constructs, allowing for further examination of the structural relationships among the variables.

### Descriptive statistics and correlations

Descriptive statistics and Pearson correlations among the study variables are presented in **Table 1**. Subjective well-being was negatively correlated with alienation and short-video addiction, and positively correlated with self-control. Alienation was positively correlated with short-video addiction and negatively correlated with self-control. Self-control was also negatively correlated with short-video addiction. Overall, the pattern of correlations was consistent with the proposed mediation model, suggesting that higher subjective well-being was associated with lower alienation, stronger self-control, and lower levels of short-video addiction.

**Table 1:** Descriptive statistics and correlations among study variables.

Variables	M	SD	1	2	3	4
1.Subjective well-being	9.86	1.68	1			
2.Alienation	67.18	14.37	-0.25***	1		
3.Self-control	56.65	10.95	0.43***	-0.38***	1	
4.Short video addiction	33.39	12.22	-0.29***	0.37***	-0.64***	1

*p< 0.05, **p< 0.01, ***p< 0.001.

### Testing of the chain mediation model

**Figure 2:**
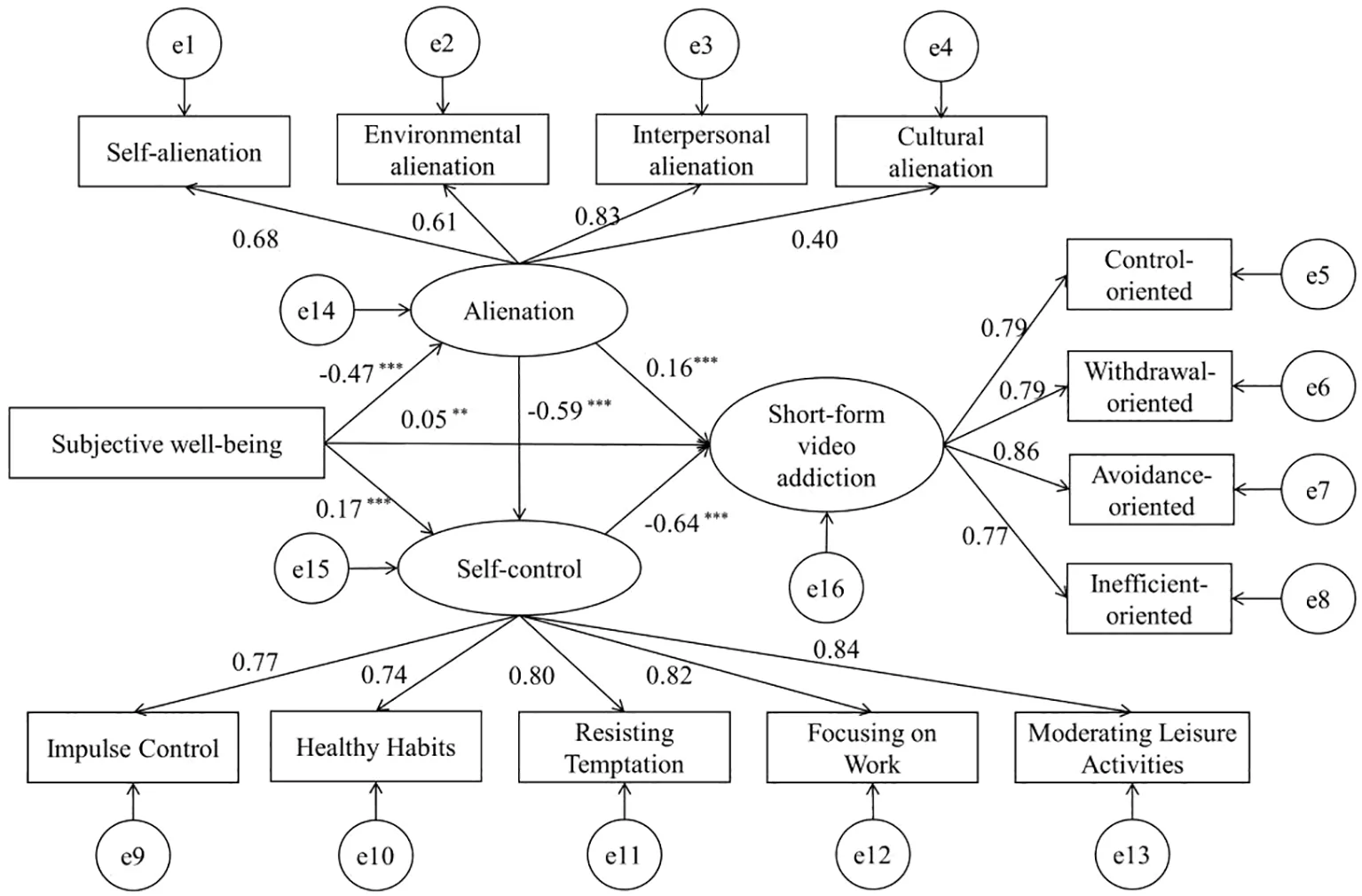
Structural equation model of the associations among subjective well-being, alienation, self-control, and short-video addiction.

To examine the proposed chain mediation model, a latent-variable structural equation model was constructed with subjective well-being as the independent variable, short-video addiction as the outcome variable, and alienation and self-control as mediating variables. The model is presented in **Figure 2**. The fit indices indicated an acceptable, though not optimal, fit to the data: χ²/df = 14.06, CFI = 0.92, RMSEA = 0.09 (90% CI [0.08, 0.09]), and SRMR = 0.07. These results suggest that the proposed model provided a reasonable representation of the observed data, with some room for improvement. To further evaluate the proposed serial mediation order, we also tested two alternative models (see **Supplementary Table 1** in the Supplementary Materials for detailed results).

As shown in **Table 2**, all hypothesized paths reached statistical significance. Subjective well-being was directly associated with short-video addiction (B = 0.08, *p* = 0.04) and was negatively associated with alienation (B = −0.62, *p* < 0.001). Subjective well-being was also positively associated with self-control (B = 0.45, *p* < 0.001). In addition, alienation was positively associated with short-video addiction (B = 0.22, *p* < 0.001) and negatively associated with self-control (B = −0.42, *p* < 0.001). Self-control was negatively associated with short-video addiction (B = −1.18, *p* < 0.001). Taken together, these results suggest that subjective well-being was associated with short-video addiction not only directly but also indirectly through the social-emotional pathway of alienation and the self-regulatory pathway of self-control.

**Table 2:** Regression coefficient analysis results for each variable.

Pathways	B	*SE*	C.R.	*P*
Subjective well-being → Short video addiction	0.08	0.04	2.09	0.04
Subjective well-being → Alienation	-0.62	0.04	-16.94	< 0.001
Subjective well-being → Self-control	0.45	0.06	6.99	< 0.001
Alienation → Short video addiction	0.22	0.04	4.84	< 0.001
Self-control → Short video addiction	-0.42	0.02	-18.41	< 0.001
Alienation → Self-control	-1.18	0.07	-17.99	< 0.001

The significance of the indirect effects was tested using a bootstrap procedure with 5,000 resamples and 95% confidence intervals. As presented in **Table 3**, the indirect effect of subjective well-being on short-video addiction through alienation was significant, 95%CI [−0.20, −0.07]. The indirect effect through self-control was also significant, 95%CI [−0.26, −0.12]. In addition, alienation significantly mediated the association between subjective well-being and self-control, 95%CI [0.60, 0.87], and self-control significantly mediated the association between alienation and short-video addiction, 95%CI [0.41, 0.59]. Finally, the serial indirect effect of subjective well-being on short-video addiction through alienation and self-control was significant, 95%CI [−0.38, −0.24]. These findings support the proposed chain mediation model, suggesting that subjective well-being was associated with short-video addiction through both separate and sequential indirect pathways involving alienation and self-control.

**Table 3:** Mediating effects and 95% confidence intervals estimated by bootstrap methods for each indirect effect.

Path	Indirect effect estimate	Lower bound	Upper bound
Subjective well-being → Alienation → Short-video addiction	-0.13	-0.20	-0.07
Subjective well-being → Self-control → Short-video addiction	-0.19	-0.26	-0.12
Subjective well-being → Alienation → Self-control	0.72	0.60	0.87
Alienation → Self-control → Short-video addiction	0.49	0.41	0.59
Subjective well-being → Alienation → Self-control → Short-video addiction	-0.30	-0.38	-0.24

## Discussion

The present study examined the psychological pathways underlying the association between subjective well-being and short-video addiction among college students, with particular attention to the mediating roles of alienation and self-control. The findings supported an integrated social-emotional and self-regulatory account. Higher subjective well-being was associated with lower alienation and stronger self-control, which were in turn related to lower levels of short-video addiction. This pattern is consistent with the social compensation model, which suggests that individuals with lower well-being and insufficient offline emotional support may be more likely to seek compensation in online environments (12). It also aligns with self-control theory, which emphasizes that negative social-emotional experiences, such as alienation, may undermine regulatory resources and weaken individuals’ capacity to resist immediate rewards (40). In the context of short-video platforms, stronger self-control may be related to students regulate viewing time, resist continuous content streams, and reduce excessive engagement (41). These findings suggest that short-video addiction among college students may be better understood as part of a broader psychological process involving both social-emotional disconnection and impaired self-regulation, rather than as a direct consequence of low well-being alone.

### Subjective well-being and short-video addiction

The present study found that subjective well-being was negatively associated with short-video addiction among college students. This finding is consistent with the social compensation model, which suggests that individuals who experience lower well-being, limited emotional support, or dissatisfaction in offline life may be more likely to seek compensation in online environments (42). In the context of short-video use, students with lower subjective well-being may turn to short-video platforms for immediate pleasure, emotional relief, or temporary escape from academic and interpersonal stress. Because short-video content is highly accessible, emotionally rewarding, and continuously updated, such compensatory use be associated with an increased likelihood of the likelihood of excessive and poorly controlled engagement (29).

Conversely, students with higher subjective well-being may have more stable emotional resources, stronger real-life engagement, and greater perceived social support. These psychological resources may be associated with a reduced need the need to rely on short-video platforms as a primary means of mood regulation or social compensation (4, 43). From this perspective, subjective well-being may function as a protective psychological correlate of problematic short-video use. However, given the cross-sectional nature of the present study, this association should be interpreted cautiously; higher subjective well-being may be linked to lower short-video addiction, but causal direction cannot be firmly established. The finding highlights the importance of considering general well-being when considering why some college students are more vulnerable to short-video addiction.

### Alienation as a mediating pathway

The present study found that alienation mediated the association between subjective well-being and short-video addiction. This finding is consistent with prior research showing that alienation is closely related to problematic Internet use and other maladaptive online behaviors (44). One possible explanation is that students with higher subjective well-being tend to experience stronger emotional security, more supportive interpersonal relationships, and greater integration into their social environment. These resources may be associated with reduced feelings of loneliness, emptiness, and disconnection, thereby lowering the likelihood that students will turn to short-video platforms as a compensatory source of emotional relief.

By contrast, students with lower subjective well-being may be more vulnerable to alienation. When individuals feel disconnected from others or unable to obtain sufficient emotional support in real life, short-video platforms may provide an easily accessible means of distraction, mood regulation, and temporary social compensation. However, repeated reliance on short-video content may further reduce real-life engagement and reinforce a sense of psychological distance from others. This interpretation is consistent with evidence that higher alienation is associated with internalizing problems and Internet-related addictive behaviors (45), whereas lower alienation and greater social integration are linked to more adaptive psychological functioning (46, 47). Thus, alienation may represent a social-emotional mechanism through which lower subjective well-being is associated with greater vulnerability to short-video addiction.

### Self-control as a mediating pathway

The present study found that self-control mediated the association between subjective well-being and short-video addiction among college students. This finding is consistent with the strength model of self-control, which emphasizes the role of self-regulatory resources in guiding goal-directed behavior and resisting immediate temptations (48). Students with higher subjective well-being may have more positive emotional experiences and greater psychological resources, which may support self-regulation, behavioral planning, and long-term goal pursuit. In contrast, lower subjective well-being is often accompanied by more negative affect and psychological distress, which may consume regulatory resources and weaken individuals’ capacity to inhibit impulsive media use (30, 31, 49).

This self-regulatory pathway is particularly relevant in the context of short-video platforms, as their design features place unique demands on users’ self-regulatory capacity —making self-control a critical factor in understanding vulnerability to short-video addiction (50). College students with stronger self-control may be better able to limit their viewing time, resist the pull of continuous content streams, and maintain a balance between online entertainment and academic or daily responsibilities (51). By contrast, students with lower self-control may be more susceptible to the immediate gratification provided by short videos and may find it more difficult to stop using them once engaged (24). Thus, self-control may represent a key self-regulatory mechanism through which subjective well-being is associated with short-video addiction. This finding suggests that problematic short-video use is not only related to students’ emotional well-being but also to their capacity to potentially translate psychological resources into effective behavioral regulation.

This pattern can be better understood by examining the unique features of short-video platforms. Unlike generalized Internet use, short-video platforms are characterized by algorithm-driven content delivery, endless scrolling, immediate emotional rewards, and low-cost entertainment (10, 13). These features may help explain why students with lower self-control are especially vulnerable to short-video addiction. Algorithmic recommendations create highly engaging content streams that are difficult to disengage from (50); endless scrolling removes natural stopping points, making it harder for users to regulate viewing time; rapid emotional rewards reinforce habitual checking, particularly for those with strong immediate gratification tendencies (29); and low cognitive cost lowers the threshold for impulsive engagement (27). Together, these platform characteristics place continuous demands on users’ self-regulatory resources, making self-control particularly critical in the context of short-video addiction.

### Serial mediation through alienation and self-control

The present study found a significant serial mediation pathway linking subjective well-being, alienation, self-control, and short-video addiction. This finding suggests that subjective well-being is associated with short-video addiction not only through separate social-emotional and self-regulatory pathways, but also through a sequential process in which alienation and self-control operate together. Students with higher subjective well-being may experience stronger emotional security, more stable interpersonal connections, and a greater sense of belonging, which may reduce feelings of loneliness, emptiness, and psychological disconnection. Lower alienation, in turn, may be associated with self-regulatory resources and support more effective control over short-video use.

This pathway is consistent with the social compensation model, which suggests that individuals with lower well-being and unmet emotional needs may be more likely to seek compensation through online media (42, 47). Alienation may represent a persistent negative social-emotional experience that consumes psychological resources and weakens self-regulation, a view consistent with the limited-resource model of self-control (40). When self-control is weakened, students may find it more difficult to regulate viewing time, resist algorithmically recommended content, and disengage from the immediate rewards provided by short-video platforms. This interpretation also aligns with the dual-component theory of self-control, which emphasizes the importance of both proactive regulation and inhibition in preventing maladaptive behaviors (52). Prior studies have similarly shown that lower self-control is closely associated with addictive online behaviors (53, 54).

The serial mediation finding provides a more integrated account of how subjective well-being is related to short-video addiction among college students. Rather than indicating a simple direct link, the findings suggest a more nuanced pathway in which lower subjective well-being is associated with greater social-emotional disconnection, weakened self-regulatory capacity, and greater vulnerability to excessive short-video use. This interpretation may extend existing research by integrating the social compensation perspective with self-control theory and suggesting the joint roles of alienation and self-control in problematic short-video use.

### Theoretical and practical implications

The present study has several theoretical and practical implications. Theoretically, the findings extend research on short-video addiction by identifying alienation and self-control as two psychological pathways linking subjective well-being to problematic short-video use among college students. Rather than focusing only on the direct association between well-being and addictive media use, this study suggests a more integrated mechanism involving both social-emotional disconnection and self-regulatory capacity. In doing so, the findings contribute to the application of the social compensation model in the context of short-video addiction and further suggest that self-control theory may be relevant to understanding why some students are more vulnerable to excessive short-video use.

Practically, the findings suggest that interventions for short-video addiction among college students should not focus solely on restricting media use or providing psychological counseling. Instead, universities could adopt a more comprehensive approach that addresses students’ social and emotional needs. For instance, universities can create more offline social opportunities—such as interest-based clubs, team-building activities, and peer mentoring programs—to help students build meaningful interpersonal connections and reduce reliance on short videos for social compensation. Improving students’ sense of campus belonging, through orientation programs, dormitory activities, and inclusive campus events, may also reduce feelings of alienation and disconnection, which emerged as a key pathway linking well-being to addictive use in our model. Moreover, universities could guide students to develop healthier digital routines by incorporating digital literacy education into orientation or mental health curricula, teaching students how to recognize early signs of problematic use, set time limits, and differentiate between purposeful and passive browsing. These strategies align with the three pathways identified in our study—enhancing well-being, reducing alienation, and strengthening self-control—and can be feasibly implemented within existing university mental health and student support systems (51, 55). Such efforts may be particularly beneficial for students with low well-being, high alienation, or weak self-control.

## Limitations and future directions

Several limitations should be noted. First, all variables were assessed using self-report questionnaires, which may be affected by social desirability, recall bias, and common method variance. Future studies should incorporate multiple sources of data, including interview data, peer or teacher reports, and objective indicators of short-video use such as usage duration, frequency, and content type. Second, the cross-sectional design limits causal interpretation and does not allow firm conclusions about the temporal ordering among subjective well-being, alienation, self-control, and short-video addiction. Longitudinal, diary, cross-lagged, or experimental designs are needed to further examine these relationships over time. Third, the sample was drawn from eight universities in Hunan Province, which may limit the generalizability of the findings. Future research should include more diverse samples across regions, university types, academic years, and disciplinary backgrounds, and further examine whether the proposed model differs by gender, major, family background, or patterns of short-video use. Fourth, subjective well-being was treated as a unidimensional construct in this study. However, it comprises both cognitive (life satisfaction) and affective components (56), which may have differential associations with alienation, self-control, and short-video addiction. Future research could examine whether these components operate through distinct pathways. Fifth, the present model focused primarily on individual psychological factors; future research could also incorporate environmental and platform-level variables (e.g., peer norms, academic pressure, recommendation algorithms) to provide a more comprehensive understanding of short-video addiction.

## Conclusion

This study examined the association between subjective well-being and short-video addiction among college students and further explored the mediating roles of alienation and self-control. The findings showed that subjective well-being was negatively associated with short-video addiction, and that alienation and self-control served as both independent and sequential mediating pathways. Specifically, lower subjective well-being was associated with greater alienation, which was further related to weaker self-control and higher levels of short-video addiction. These results suggest that problematic short-video use among college students may be understood not only as a behavioral issue, but also as a psychological process involving social-emotional disconnection and impaired self-regulation. By integrating the social compensation perspective with self-control theory, this study provides a more comprehensive account of the mechanisms linking well-being to short-video addiction and suggests the importance of enhancing subjective well-being, reducing alienation, and strengthening self-control in prevention and intervention efforts.

## Data Availability

The raw data supporting the conclusions of this article will be made available by the authors, without undue reservation.

## Supplementary material

Supplementary Table 1 Fit indices for alternative serial mediation models comparing the hypothesized model with alternative ordering of mediators and a parallel mediation model.
