# Supplementary material for: Linking subjective well-being to short-video addiction among college students: the serial mediating roles of alienation and self-control
Source: Front Psychiatry. 2026 Aug 20;17:1892672. doi: 10.3389/fpsyt.2026.1892672 (PMC13539139; doi:10.3389/fpsyt.2026.1892672)
Supplement: Supplementary file 1 [file Table1.docx]

**Supplementary Materials**

**Table S1**

*Fit Indices for Alternative Models*

| Model | χ² | df | χ²/df | CFI | RMSEA | AIC |
| --- | --- | --- | --- | --- | --- | --- |
| Subjective well-being → Alienation → Self-control → Short video addiction | 4094.62 | 288 | 14.22 | 0.93 | 0.04 | 4358.62 |
| Subjective well-being → Self-control → Alienation → Short video addiction | 4094.62 | 288 | 14.22 | 0.93 | 0.04 | 4358.62 |
| Parallel mediation | 5466.91 | 292 | 18.72 | 0.90 | 0.05 | 5722.91 |
